# L1_0_-FeNi films on Au-Cu-Ni buffer-layer: a high-throughput combinatorial study

**DOI:** 10.1038/s41598-018-34296-9

**Published:** 2018-10-29

**Authors:** G. Giannopoulos, G. Barucca, A. Kaidatzis, V. Psycharis, R. Salikhov, M. Farle, E. Koutsouflakis, D. Niarchos, A. Mehta, M. Scuderi, G. Nicotra, C. Spinella, S. Laureti, G. Varvaro

**Affiliations:** 1 Institute of Nanoscience and Nanotechnology, NCSR Demokritos, Athens, Greece; 2 Università Politecnica delle Marche, Dipartimento SIMAU, Via Brecce Bianche 12, Ancona, 60131 Italy; 3 Faculty of Physics and Center for Nanointegration (CENIDE), University of Duisburg-Essen, 47057 Duisburg, Germany; 4 Zavoisky Physical-Technical Institute, FRC Kazan Scientific Center of RAS, 420029 Kazan, Russian Federation; 5 Center for Functionalized Magnetic Materials (FunMagMa), Immanuel Kant Baltic Federal University, Kaliningrad, Russian Federation; 6 SLAC National Accelerator Laboratory- Stanford University, Menlo Park, California, USA; 7 IMM-CNR, VII strada 5, 95121 Catania, Italy; 8 Istituto di Struttura della Materia, CNR, Monterotondo Scalo, Roma, Italy

## Abstract

The fct L1_0_-FeNi alloy is a promising candidate for the development of high performance critical-elements-free magnetic materials. Among the different materials, the Au-Cu-Ni alloy has resulted very promising; however, a detailed investigation of the effect of the buffer-layer composition on the formation of the hard FeNi phase is still missing. To accelerate the search of the best Au-Cu-Ni composition, a combinatorial approach based on High-Throughput (HT) experimental methods has been exploited in this paper. HT magnetic characterization methods revealed the presence of a hard magnetic phase with an out-of-plane easy-axis, whose coercivity increases from 0.49 kOe up to 1.30 kOe as the Au content of the Cu-Au-Ni buffer-layer decreases. Similarly, the out-of-plane magneto-crystalline anisotropy energy density increases from 0.12 to 0.35 MJ/m^3^. This anisotropy is attributed to the partial formation of the L1_0_ FeNi phase induced by the buffer-layer. In the range of compositions we investigated, the buffer-layer structure does not change significantly and the modulation of the magnetic properties with the Au content in the combinatorial layer is mainly related to the different nature and extent of interlayer diffusion processes, which have a great impact on the formation and order degree of the L1_0_ FeNi phase.

## Introduction

The ferromagnetic equiatomic FeNi alloy with a face-centered tetragonal (fct) L1_0_-type structure, also known as *tetrataenite*, is a promising candidate for the replacement of high-anisotropy magnetic materials containing rare-earths and critical elements^1–5^ due to its excellent intrinsic magnetic properties, such as large saturation magnetization (~1.6 T), high uniaxial magneto-crystalline anisotropy (MCA~1 MJ/m^3^), fairly high Curie temperature (up to 550 °C) and low magnetization damping constant^6–8^. The fabrication of the L1_0_-FeNi phase is extremely challenging due to the low atomic mobility below the chemical order/disorder transition temperature (~320 °C)^9^ that kinetically limits the formation of the L1_0_ phase. This FeNi phase is naturally found in meteorites, where it forms over millions of years in extreme temperature/pressure conditions^6^. Different strategies have been proposed to artificially obtain the tetrataenite phase, including deposition of alternate Fe and Ni monoatomic layers^10–14^, irradiation with neutrons or high energy electrons^15,16^, addition of a third element^7,16^ or by exploiting the epitaxial strain induced by suitable templates in both thin films^13,17–20^ and nanoparticles^21^ systems.

The origin of the large magneto-crystalline anisotropy in L1_0_-FeNi and its relation to the strain effect has been extensively discussed. According to theoretical calculations, the MCA in L1_0_-FeNi systems can be predominantly attributed to the constituent Fe atoms and increases by decreasing the *a*/*c* axial ratio^22^ (*a* and *c* corresponding to the cubic in-plane and out-plane lattice constant). The predicted trend for the MCA energy is also in line with experimental studies, where tetragonal L1_0_-ordered FeNi films were stabilized using the alternate deposition of Fe and Ni monatomic layers^23^. Furthermore, an extensive theoretical work has been done about phase stability^24^, phase transformation, segregation effects^25^ and coercivity improvement through nano-structuring in FeNi alloys^26^. Theoretical works support that structural parameters of the FeNi alloy significantly depend on the Fe/Ni ratio as the Young’s modulus of the bcc Fe increases by enhancing the concentration of nickel^27^.

By following the Bain path^28^, metastable tetragonal phases can be stabilized by straining the unit cell^29^. For the FeNi alloy, the tetragonal distortion can be induced via coherent growth of the film on different substrates (e.g. MgO (100)^17^ or Cu (001)^18^) or buffer-layers (Cu^13,19^, Cu-Ni^20^ and Au-Cu-Ni^8,11,14^). Among them, the Au-Cu-Ni alloy has resulted very promising; however, a detailed investigation of the effect of the buffer-layer composition on the formation of the tetragonal phase is still missing.

To speed-up the search of the best Au-Cu-Ni composition, a combinatorial approach based on High-Throughput (HT) experimental methods has been exploited in this paper. HT techniques aim at fabricating materials libraries by creating ternary or quaternary compounds as candidate structures. On such a library, a well-defined set of materials with different chemical compositions is deposited in a single run, that means at a fraction of a time compared to conventional approaches. Data mining techniques^30^ are then used for a robust and effective materials analysis^31^ to reinforce and accelerate the experimental search by identifying the most promising alloys’ stoichiometry.

In this work, a Cu-Au-Ni compositional spread library was deposited as a buffer-layer by using a Combinatorial Sputtering Technique^32^ to tune the lattice constant and tailor the lattice mismatch with a FeNi film deposited on top in order to determine the best stoichiometry promoting the L1_0_-ordering. Magneto-Optic Kerr Effect (MOKE) analysis was used for a first screening of the FeNi magnetic properties. X-ray Fluorescence (XRF), Wide-angle X-ray Scattering (WAXS), Scanning Transmission Electron Microscopy (STEM), Energy Dispersive X-ray (EDX) microanalysis and Ferromagnetic Resonance (FMR) measurements were performed to investigate the structural, chemical and magnetic properties of selected samples.

## Results and Discussion

**Figure 1:**
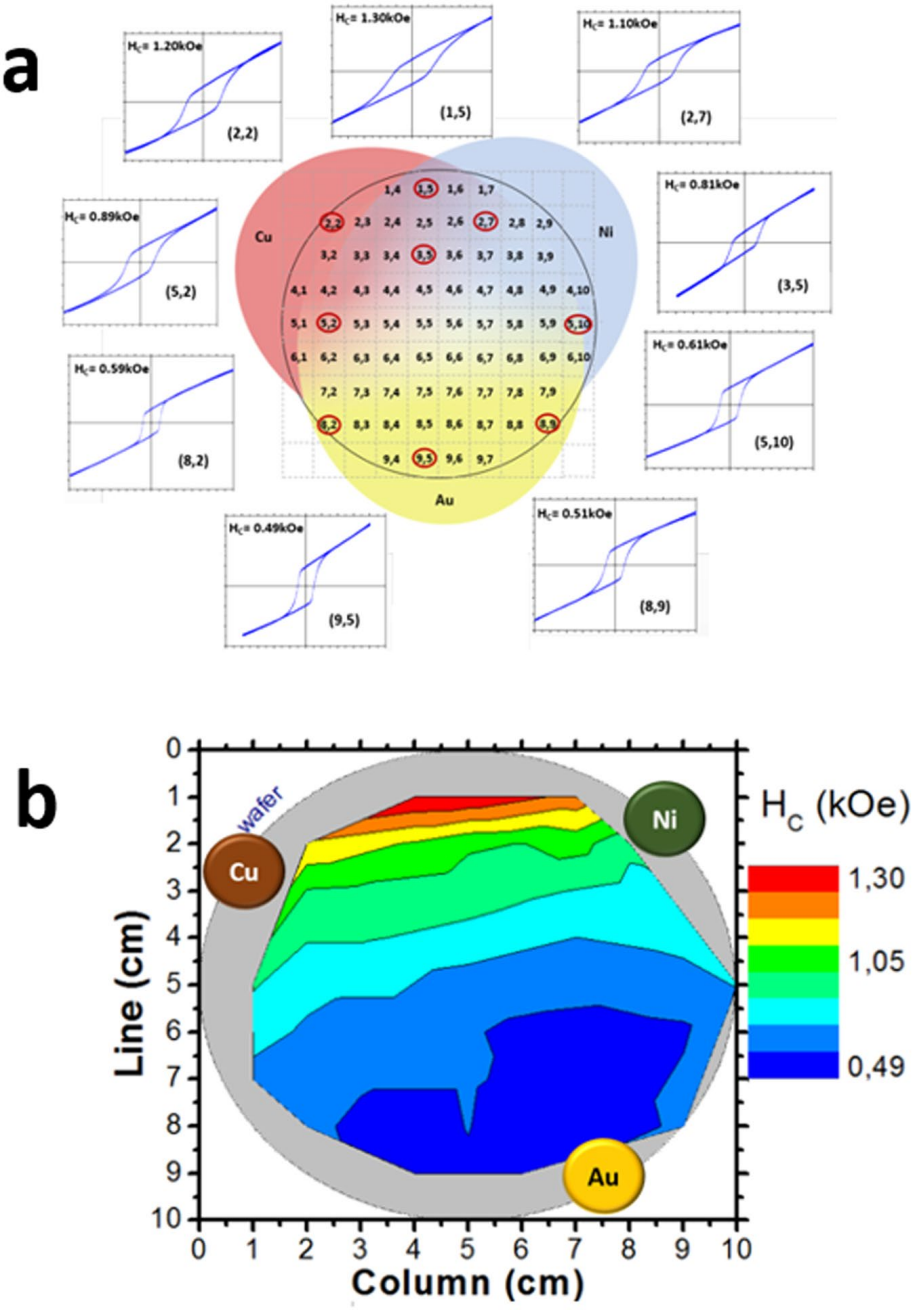
(**a**) Representative room temperature polar field-dependent magnetization loops showing the systematic change of the coercivity (H_C_) with the Au-Cu-Ni stoichiometry. (**b**) Color-coded coercive field diagram; the numbers (X, Y) identify the measured areas on the wafer corresponding to different Au-Cu-Ni compositions. The applied magnetic field range of the hysteresis loops is ±5 kOe.

Figure 1a reports on representative polar field-dependent magnetization loops collected at room temperature from selected areas on the wafer, corresponding to different nominal Au-Cu-Ni compositions measured by XRF (data provided in Supplementary Materials Section, Figure S1a–c). All the loops present a hysteretic feature superimposed to a linear *M*(*H*) contribution. The hysteretic behavior suggests the presence of a hard magnetic phase with an out-of-plane magnetic anisotropy. The linear contribution to the magnetization loops can be ascribed either to hard magnetic regions with the easy-axis pointing along different directions with respect to the normal to the film surface or to the presence of soft magnetic grains whose anisotropy lies in the film plane due to the demagnetizing fields.

As clearly shown in Fig. 1a, and schematically reported in Fig. 1b, the out-of-plane coercivity, extracted from the maximum in susceptibility evaluated by the field derivative of the hysteresis loop, increases by reducing the Au content from *H*_*C*_ = 0.49 kOe (on Au_18.2_Cu_40.0_Ni_38.5_) up to 1.3 kOe (on Au_13.4_Cu_47.5_Ni_44.7_).

The significant change of *H*_*C*_ cannot be attributed to the film microstructure, as the surface morphology of the film, measured by AFM (data provided in Supplementary Materials Section, Figure S2) remains almost unchanged across the wafer. Moreover, being the MOKE technique only sensitive to the magnetization within the skin depth region of a film, typically 20–40 nm in most metals^33^, the performed measurements are only sensitive to the FeNi layer (nominal thickness: 40 nm).

To disclose the effect of the buffer-layer composition on the magnetic properties of the FeNi layer, a thoroughly investigation of both the structural and microstructural properties have been performed on three selected samples with different out-of-plane coercivity (varying from 1.30 kOe in the high-coercivity region down to 0.49 kOe in the low-coercivity region) corresponding to three different nominal compositions of the combinatorial layer (Au_13.4_Cu_47.5_Ni_44.7_, Au_14.8_Cu_45.6_Ni_39.6_ and Au_18.2_Cu_40.0_Ni_38.5_ respectively). Hereafter the three samples will be referred as *H*-FeNi, *M*-FeNi and *L*-FeNi, where *H*, *M* and *L* stand for high, medium and low coercivity, respectively.

**Figure 2:**
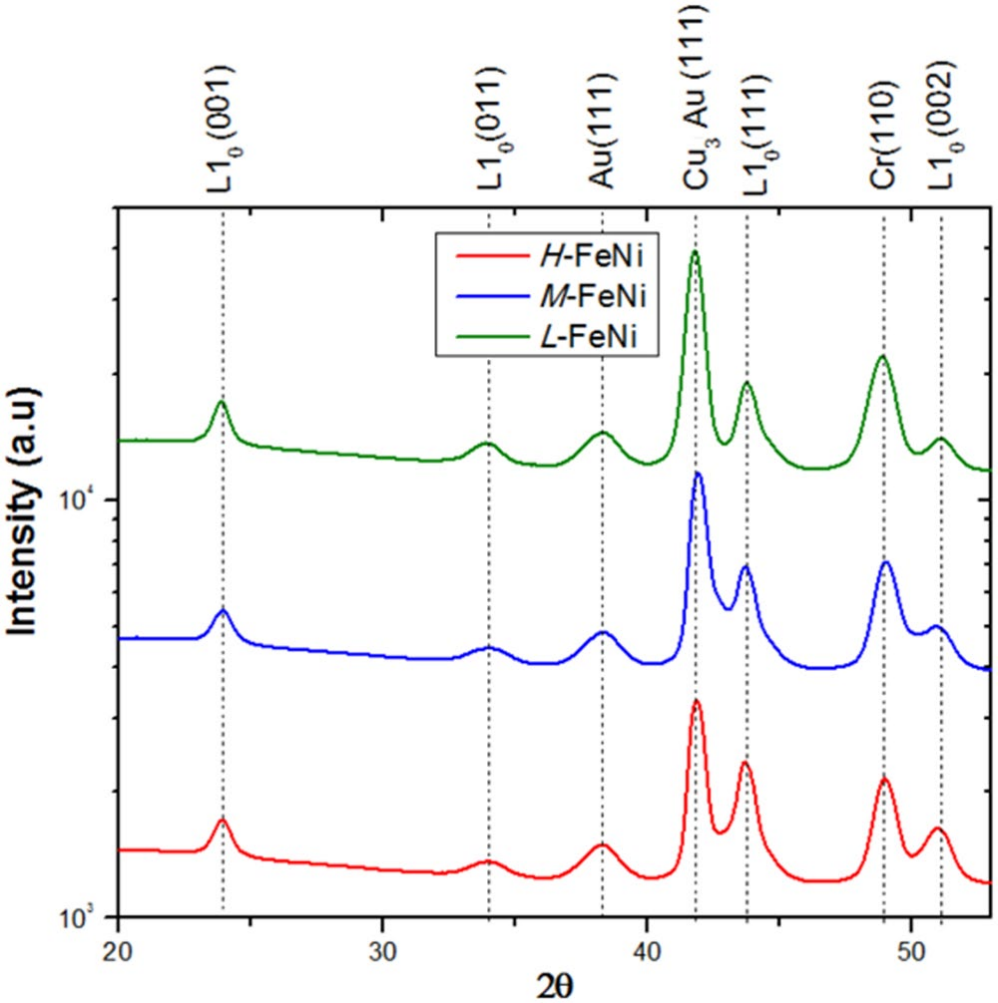
WAXS diffractograms of samples *H*-FeNi, *M*-FeNi and *L*-FeNi corresponding to different regions of the compositional spread materials library. The dashed lines indicate the reference peak positions for the different phases.

Wide-angle X-ray Scattering (WAXS) has been employed to examine the structural properties of the samples (Fig. 2). Diffraction peaks attributed to the seed and Au capping layers are indicated along with the reflections corresponding to the L1_0_-FeNi phase^34,35^. The WAXS measurements were collected in grazing incidence geometry where the angle of incidence is fixed while the diffracted angle is varied (see the Supplementary Materials Section, Figs S3 and S4). In our diffraction geometry, the observed (001) L1_0_-FeNi peak probes grains for which the c-axis (corresponding to the magnetic easy-axis) forms a relatively small angle of 4° with respect to the perpendicular to the film surface thus justifying the observed hysteretic behaviour measured by the polar MOKE. The FeNi (111) reflection peak cannot be univocally assigned to the L1_0_-phase (a contribution from the chemical disordered FeNi phase cannot be excluded); however, the possible presence of (111) and (011) L1_0_-FeNi grains would also give rise to a contribution to the magnetic behaviour along the normal to the film plane, being the corresponding c-axis, in both the cases, tilted away from the film surface (see the Supplementary Materials Section). Moreover, the presence of such hard magnetic grains with a tilted easy-axis along with a fraction of soft magnetic grains, can explain the observed linear contribution to the polar MOKE magnetization loops.

It must be stressed the absence of peaks due to the CuAuNi combinatorial layer, suggesting a lattice matching between the Cu_3_Au seed-layer and the overlying combinatorial layer independently of its composition. Furthermore, the presence of a single diffraction peak related to the seed-layer indicates that the chemical ordered Cu_3_Au phase (ICDD n° 351357) is highly textured.

**Figure 3:**
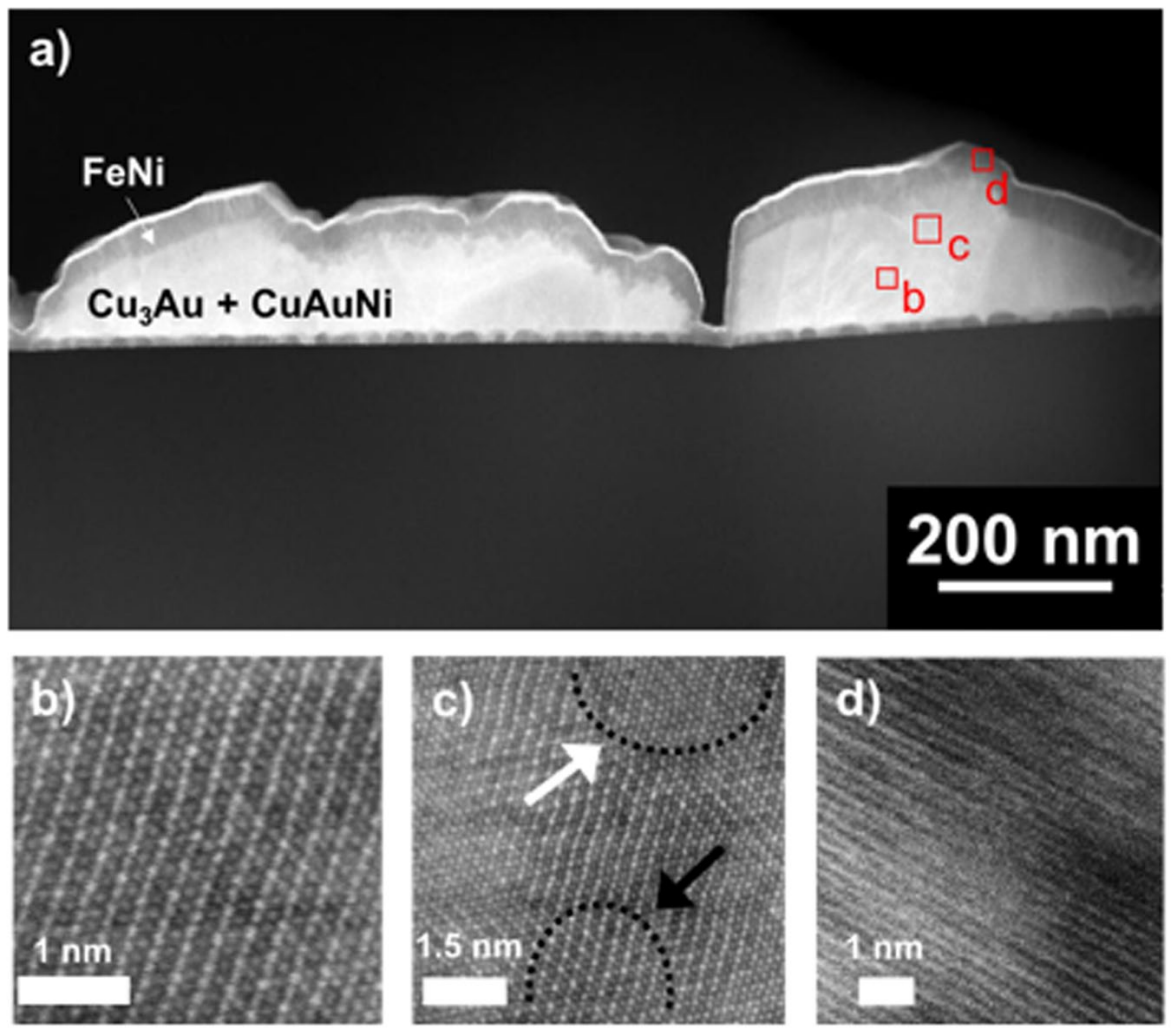
H-FeNi Sample. (**a**) HAADF-STEM image. (**b**), (**c**) and (**d**) HR-HAAFD STEM images of selected regions of the island in the <111> Cu_3_Au zone axis.

To further investigate the structure of the samples, STEM analyses were conducted in the high- and low-coercivity regions of the wafer (samples *H*-FeNi and *L*-FeNi, respectively). Fig. 3a shows a high-angle annular dark field (HAADF) STEM image of sample *H*-FeNi. The film shows an islands structure (in agreement with the AFM images), and it is not possible to distinguish the Cu_3_Au seed-layer from the combinatorial Au-Cu-Ni layer inside the islands. To investigate the nature of the combinatorial layer, atomic resolution HAADF-STEM (HR-HAADF STEM) observations were performed. In detail, Fig. 3b shows the atomic structure of the Cu_3_Au phase in the <111> zone axis. Due to the typical Z-contrast (atomic number contrast) of the technique, gold atoms appear brighter than copper ones. A representative portion of the combinatorial layer is shown at the top side of Fig. 3c (circled area, white arrow). Atoms (white spots) have all the same contrast, revealing the absence of chemical order, and their distribution is compatible with a face centered cubic (fcc) phase in the <111> zone axis and lattice parameter *a* = (3.69 ± 0.04) Å. The transition region between the Cu_3_Au layer (circled area, dark arrow) and the combinatorial layer is also visible in Fig. 3c. Atomic planes extend from one phase to the other without interruption (area between dark dotted lines) as occurs in epitaxial growth mechanisms. Considering the local nature of HR-HAADF STEM observations, the term “local epitaxy” will be used to describe this lattice matching in the rest of the paper. The FeNi layer is clearly visible at the top of the islands in Fig. 3a and it has a thickness of about 40 nm, in agreement with the deposition nominal value.

A HR-HAADF STEM image of the FeNi layer is shown in Fig. 3d. The atomic planes appear as white lines and their interplanar distance d = (1.80 ± 0.01) Å is compatible with the (002) planes of the FeNi phase. Interestingly, at the top-left corner it is possible to observe white alternating lines having different contrast (thin lines between large ones). This contrast variation of the lattice planes is compatible with the chemical order of the L1_0_ FeNi phase in the [001] direction. On the other side of the image, the contrast is the same for all the lattice planes, indicating the absence of a chemical order^33^. To further confirm the presence of the ordered FeNi phase, computer simulations of the HR-HAADF STEM image were performed by using the “jems” software^36^. Results are shown in the Supplementary Materials Section (Figs S7 and S8) and confirm both the possibility to use atomic resolution Z-contrast to distinguish Fe and Ni atoms and the presence of the ordered FeNi phase associated to the alternating lines having different contrast. Experimental and simulation results suggest the contemporary presence of chemical ordered and disordered phases in the FeNi layer.

**Figure 4:**
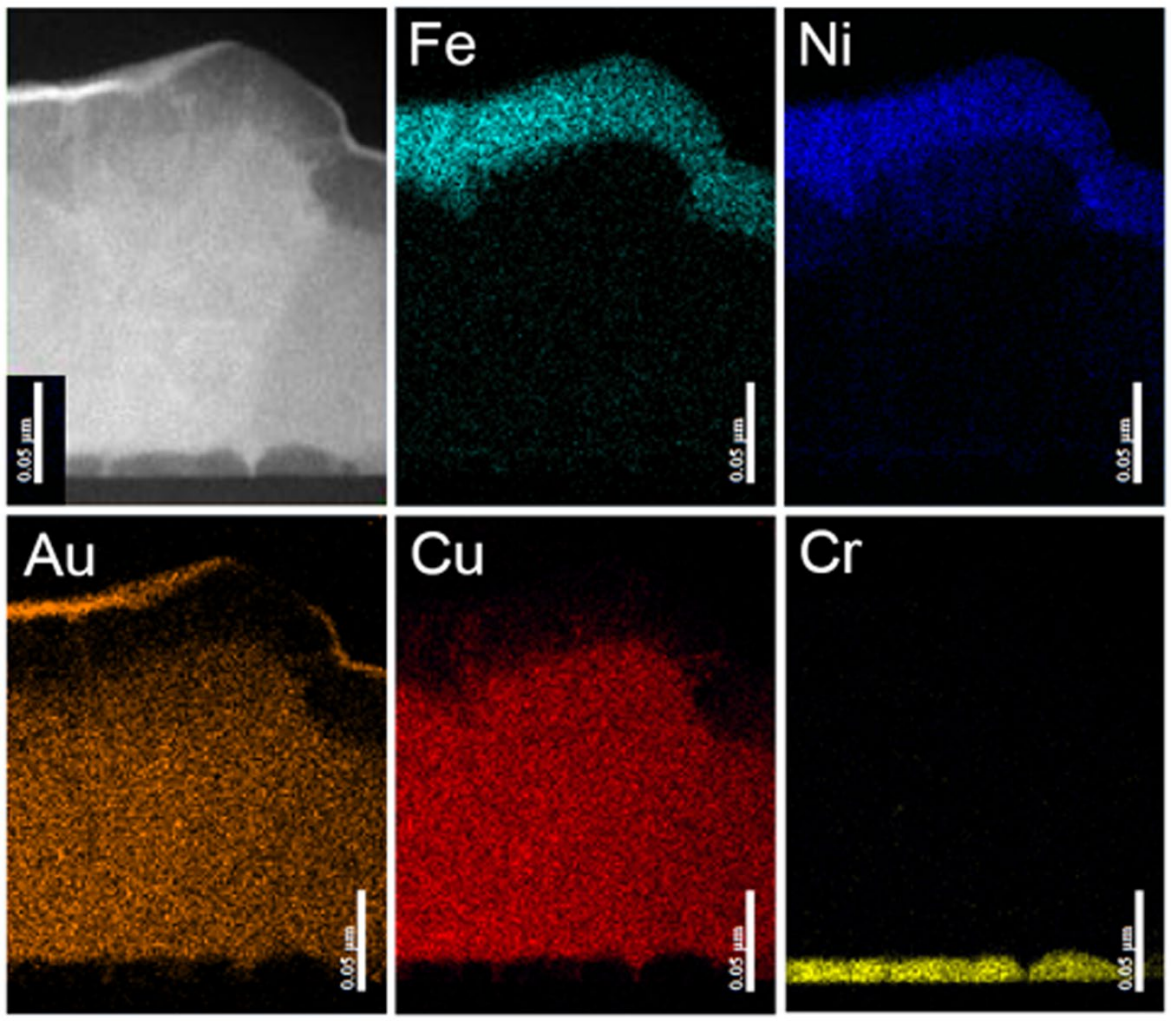
H-FeNi Sample. EDX compositional maps of the island area reported on the top-left corner.

EDX analysis was performed on a portion of the island at the right side of Fig. 3a. Distribution maps of the different elements and the island region investigated are shown in Fig. 4. From these images, it is possible to identify the different layers and the corresponding thicknesses are clearly inferable. It is important to note the presence of a slight diffusion of atomic species from the combinatorial to the FeNi layer. In particular, the brighter strips visible in the FeNi layer are associated to the presence of copper and gold atoms.

**Figure 5:**
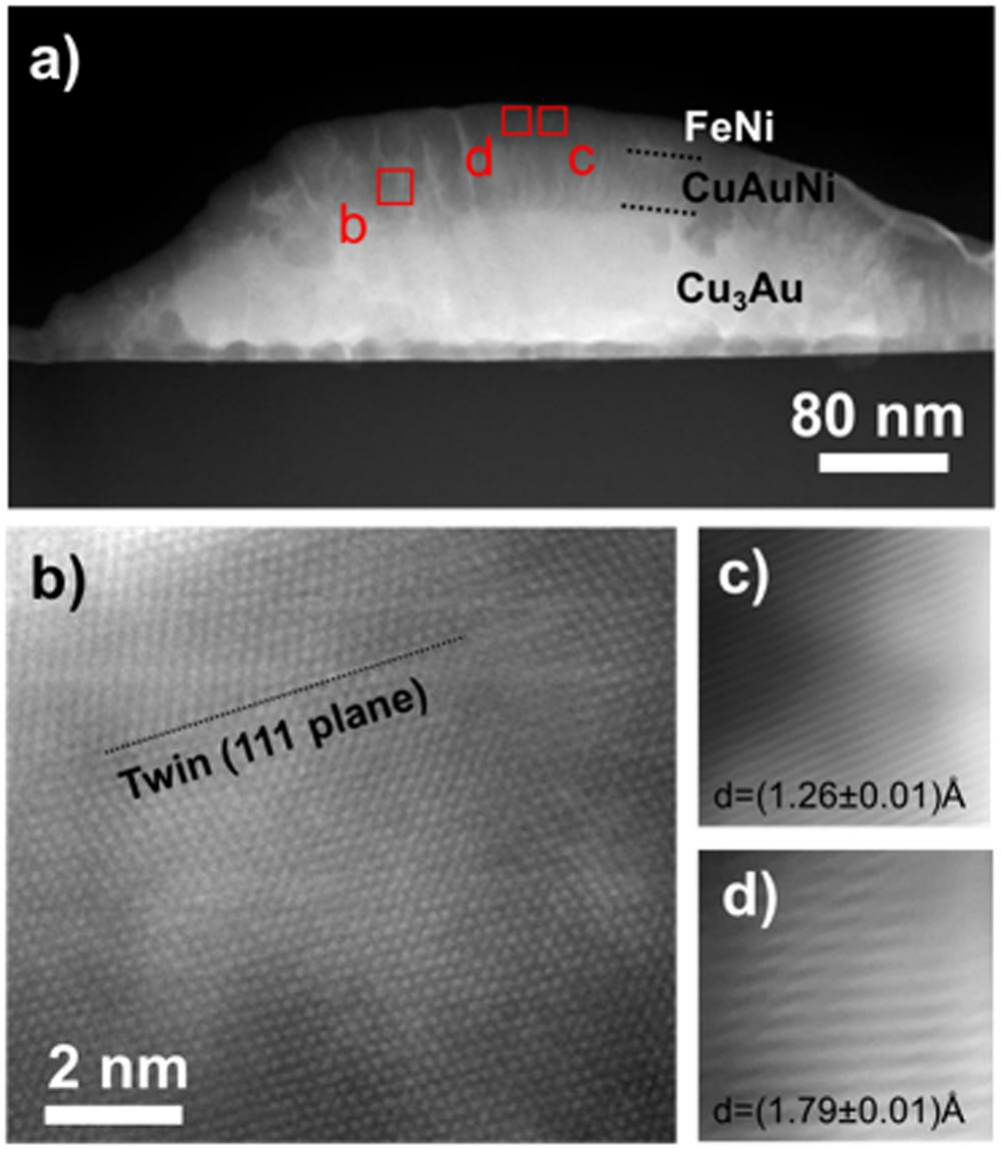
Sample L-FeNi sample. (**a**) HAADF-STEM image. (**b**), (**c**) and (**d**) HR-HAADF STEM images of the selected regions of the island in the <110> Cu_3_Au zone axis.

A HAADF-STEM image of sample *L*-FeNi is shown in Fig. 5a. Although the film is still composed of islands, however the inner structure results different. In particular, it is quite difficult to distinguish the FeNi layer from the combinatorial phase. The absence of contrast in transmission electron microscopy (data provided in Supplementary Materials Section, Figure S5) and HAADF-STEM mode suggests a local epitaxial growth between the two layers and a quite similar average atomic number. The structure of the combinatorial layer is evidenced by HR-HAADF STEM observations. As for the previous sample, the layer presents a face centred cubic (fcc) structure with a slightly smaller lattice parameter *a* = (3.55 ± 0.04) Å. Fig. 4b shows a typical HR-HAADF image of the combinatorial layer taken in the <110> zone axis orientation, which reveals the presence of a {111} twin plane. The presence of these defects can be responsible for lattice planes rotation in the FeNi layer, and of the corresponding *c*-axis^37^.

**Figure 6:**
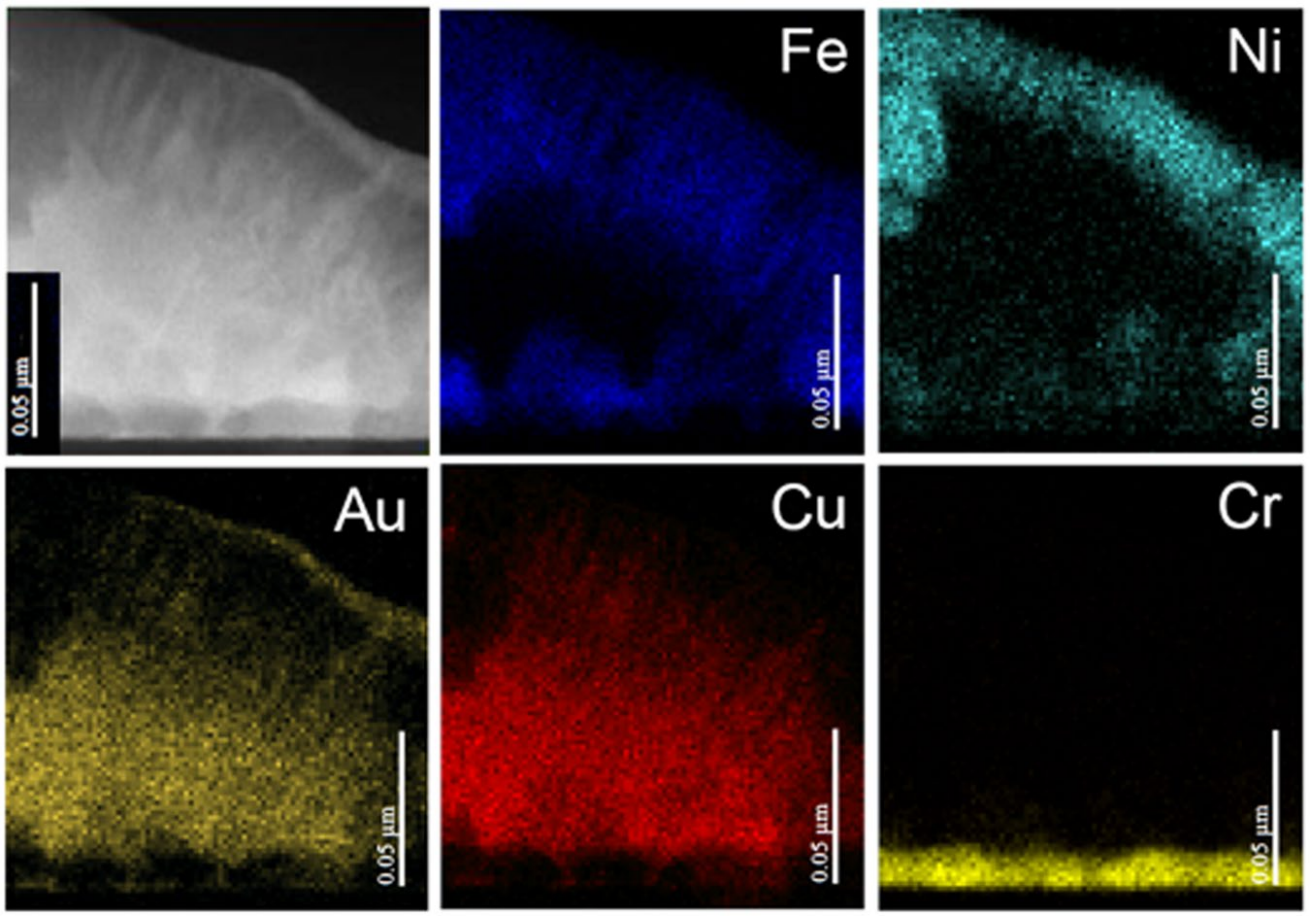
Sample L-FeNi sample. EDX compositional maps of the island area reported on the top-left corner.

HR-HAADF STEM images on the FeNi layer are reported in Fig. 5c,d. Two families of atomic planes are visible, the interplanar distances being d = (1.26 ± 0.01) Å and d = (1.79 ± 0.01) Å, compatible with the (022) and (002) planes of the FeNi phase, respectively. It must be stressed that in Fig. 5d the contrast of each visible plane is the same suggesting the absence in this specific region of the sample of any chemical order for the FeNi phase in the c-direction. However, the presence of the L1_0_-FeNi phase, as clearly shown in the WAXS spectra, cannot be excluded in other regions of the films. EDX analysis was performed on the island shown in Fig. 5a. The distribution map of the different elements and the island area investigated are shown in Fig. 6. From the EDX maps it is possible to identify the different layers and estimate their thickness. The presence of the diffusion processes from the combinatorial to the FeNi layer are clearly visible. In particular, the brighter strips, starting from the Cu_3_Au seed-layer and arriving up to the top of the FeNi layer are very rich in Au and reveal diffusion and accumulation processes of gold through the layers. A small diffusion of Cu atoms in the FeNi layer is also present. Fe and Ni atoms are also visible at the bottom of the island, but their presence is due to the specific morphology of the sample and is attributed to superposition effect in the STEM image formation, being the STEM-EDX measurements a bi-dimensional projection of a three-dimensional structure.

WAXS and STEM measurements have shown a very complex structure of the samples by varying the gold composition of the combinatorial layer. In detail, the chemical ordered L1_0_-FeNi phase is present in all the samples regardless of the combinatorial layer stoichiometry, as evidenced by WAXS spectra. Indeed, electron microscopy analyses carried out on the *H*-FeNi and *L*-FeNi regions have shown the same face centered cubic (fcc) structure for the Cu-Au-Ni layer, which grows in a locally epitaxial way on the Cu_3_Au seed-layer. Such locally epitaxial grow is suggested to cause a strain-driven diffusion of elements^38^ during the deposition, which depends on the buffer-layer composition. The nature and extent of the diffusion processes determine the amount and likely the chemical order degree of the FeNi L1_0_ phase formed in the different samples, thus leading to different magnetic properties.

**Figure 7:**
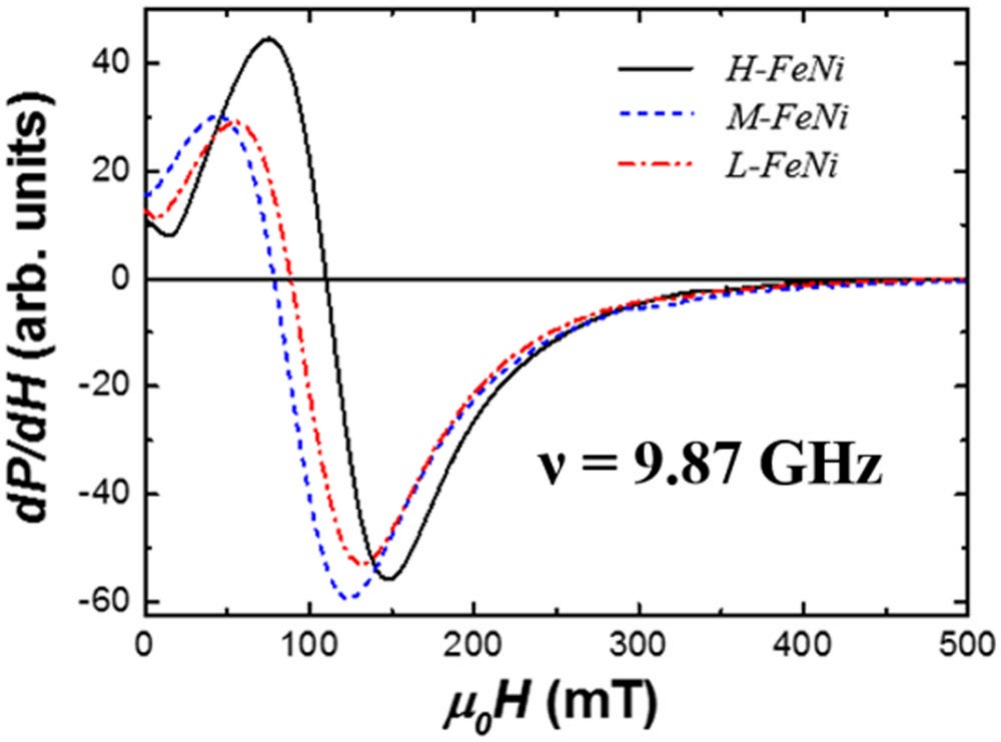
Derivative of FMR absorbed microwave power (P) as a function of the applied magnetic field for samples H-FeNi, M-FeNi and L-FeNi. All spectra were recorded at room temperature with the magnetic field applied parallel to the film plane.

To get further insight into the magnetic properties of the samples, as a function of the Au content, ferromagnetic resonance (FMR) spectra of *H*-FeNi, *M*-FeNi and *L*-FeNi were recorded with the external magnetic field applied parallel to the film plane (Fig. 7). All the spectra show resonances at magnetic fields smaller than *ω*/*γ* = 336 mT (*ω* = 2π*ν*, *ν* is the microwave frequency, *γ* = *gμ*_*B*_/ħ is the electron gyromagnetic ratio, *g* = 2.1 is the spectroscopic splitting factor assumed for the Fe_50_Ni_50_^39^), thus indicating that the magnetization easy axis preferentially lies in the film plane due to the larger contribution of magnetic shape anisotropy in FeNi films^40,41^. The in-plane angular dependent measurements do not reveal any magnetic anisotropy with axial symmetry lying in the film plane. From the analysis of the measured resonance fields (*H*_r_) in the in-plane geometry (Fig. 7) we find that the equilibrium direction of magnetization is parallel to the applied magnetic field and the resonance conditions can be described very accurately using the Kittel equation as was shown elsewhere^42^:

1 $${(\frac{\omega }{\gamma })}^{2}={\mu }_{0}^{2}{H}_{{\rm{r}}}({H}_{{\rm{r}}}+{M}_{eff}),$$

where *μ*_0_*M*_*eff*_ = *μ*_0_*fM*_*s*_ − 2*K*_2_/*M*_*s*_ is the effective magnetization. Τhe saturation magnetisation *M*_*s*_ as determined from magnetometry measurements, was found to be similar in all regions of the wafer (*μ*_0_*M*_*s*_ = 1500 ± 100 mT). A volumetric filling factor *f* = 0.97 (determined from the AFM images) was introduced in order to consider the granular structure of magnetic films^43^. The parameter *K*_2_ is used to account for the out-of-plane magneto-crystalline anisotropy energy density, which competes with the shape anisotropy of ferromagnetic films and favours the perpendicular alignment of the magnetization with respect to the film plane. In Fig. 7 it can be observed that the resonance field for the high coercive field region (*μ*_0_*H*_r_ = 110 mT) is larger than the corresponding for the low coercive field region (*μ*_0_*H*_r_ = 82 mT). This indicates that the perpendicular magnetic anisotropy (*K*_2_) in the *H*-FeNi sample is larger than in *M*-FeNi and *L*-FeNi films, in line with the MOKE results. Using the equation (1) the estimated MCA energy density moves form *K*_2_ = 0.35 ± 0.01 MJ/m^3^ for the *H*-FeNi sample to *K*_2_ = 0.12 ± 0.01 MJ/m^3^ for the *L*-FeNi sample. In any case, the obtained MCA energy densities in all the samples are by two orders of magnitude larger than the values known for the cubic Fe_50_Ni_50_ alloy^44^. The film thickness of 40 nm is large enough to exclude a significant interface contribution to the MCA^29,40^. WAXS and HR-STEM analyses reveal the presence of the ordered L1_0_-FeNi phase, which explains the relatively large MCA in all the samples. Remarkably, the MCA in the *H*-FeNi sample is comparable to the anisotropies reported for L1_0_-ordered FeNi films stabilized using the alternate deposition of Fe and Ni mono-atomic layers^10,11^. Smaller MCA in *M*-FeNi and *L*-FeNi samples suggests reduced chemical order degree of the L1_0_-FeNi^16^ phase, which can be affected by the inter-diffusion processes.

## Conclusion

A Cu-Au-Ni compositional spread library was deposited as buffer-layer to explore the effect of different compositions on the formation of the L1_0_-FeNi phase. HT magnetic characterization methods revealed the presence of a hard magnetic phase with an out-of-plane anisotropy, whose coercivity, measured along the film normal, increases from approximately 0.5 kOe up to 1.3 kOe as the Au content of the Cu-Au-Ni buffer-layer decreases. Similarly, the out-of-plane magneto-crystalline anisotropy energy density increases from 0.12 to 0.35 MJ/m^3^, such large values being related to the formation of the L1_0_ FeNi phase induced by the buffer-layer. We remarkably observed that, in the range of compositions we investigated, the layers grow in a locally epitaxial way and a complex strain-driven diffusion mechanism, which depends on the Cu-Au-Ni atomic composition, was disclosed. Consequently, the great variation of the magnetic properties along the wafer surface can be mainly attributed to a strain-driven diffusion effect, strongly dependent on the stoichiometry of the buffer-layer, which is supposed to greatly influences the amount and/or the order degree of the L1_0_-FeNi phase.

By exploiting the advantage of a combinatorial approach, our study allows providing guidelines for the preparation of the high anisotropy L1_0_-FeNi phase; indeed, the results suggest that including a thin diffusion barrier layer between the Au-Cu-Ni and the FeNi layers, by keeping the crystallographic texture while reducing or even avoiding the diffusion effect, would allow to fully exploit the potential of the Au-Cu-Ni alloy to obtain highly ordered FeNi L1_0_ thin films.

## Methods

### Material Preparation

A high-vacuum AJA Int. ATC 2200-V magnetron sputtering system with a base pressure of 5 × 10^−9^ Torr was used to deposit on a 4-inch Si (100) substrate a Cr(10 nm)/AuCu_3_(70 nm)/combi-Au-Cu-Ni/NiFe(40 nm) stack (the reported thicknesses are nominal), where the combi-Au-Cu-Ni buffer-layer is a compositional spread layer of various stoichiometries co-deposited using combinatorial sputtering. The thickness of the combinatorial was estimated at 50 nm at the center of the Si wafer. The rest of the layers have homogeneous composition and thickness. The final deposition of FeNi was performed by co-sputtering Fe and Ni to a stoichiometry of 50-50 at.%. The Cr and Cu_3_Au seed-layers were deposited at 300 °C, while the combinatorial Au-Cu-Ni and Fe_50_Ni_50_ layers were grown at 200 °C; the Ar pressure was set at 3mTorr for all the layers.

### Material Characterization

Room temperature magnetic properties were mapped across the wafer by using a High-Throughput polar magneto-optical Kerr effect (HT-MOKE) magnetometer with a maximum external magnetic field of 1.1 T. The spatial resolution, as determined from the laser spot size, is approximately 2 mm. Thin films’ structure and microstructure were investigated by Scanning Transmission Electron Microscopy (STEM) techniques. A JEOL JEM-ARM200F microscope equipped with a microanalysis system EDS Centurio was used for STEM observations and Energy Dispersive X-ray (EDX) measurements. MCA energy density of selected sample areas was determined using a conventional Bruker X-band ferromagnetic resonance (FMR) spectrometer operating at a microwave frequency of *ν* = 9.87 GHz. FMR spectra were recorded, at room temperature for different orientations of the static magnetic field within the film plane (in-plane measurements). Wide Angle X-ray scattering (WAXS) and X-ray Fluorescence (XRF) measurements have been performed at beam-line (BL) 1–5 bending magnet end station, at the Stanford Synchrotron Radiation Laboratory (SSRL/SLAC). BL1–5 is equipped with a two dimensional Rayonix 165 CCD detector for X-ray diffraction analysis and a vortex detector for paralleled XRF analysis. The X-ray energy employed during experiments was 12.7 keV and the angle of incidence was 4°.

## Electronic supplementary material

Supplementary Information
