# Supplementary material for: L10-FeNi films on Au-Cu-Ni buffer-layer: a high-throughput combinatorial study
Source: Sci Rep. 2018 Oct 29;8:15919. doi: 10.1038/s41598-018-34296-9 (PMC6206008; doi:10.1038/s41598-018-34296-9)
Supplement: Supplementary file 1 — Supplementary Information [file 41598_2018_34296_MOESM1_ESM.docx]

**L1_0_-FeNi films on Au-Cu-Ni buffer-layer: a high-throughput combinatorial study**

G. Giannopoulos^1*^, G. Barucca^2*^, A. Kaidatzis^1^, V. Psycharis^1^, R. Salikhov^3,4^, M. Farle^3,5^, E. Koutsouflakis^1^, D. Niarchos^1^, A. Mehta^6^, M. Scuderi^7^, G. Nicotra^7^, C. Spinella^7^, S. Laureti^8^ and G. Varvaro^8^

*^1^Institute of Nanoscience and Nanotechnology, NCSR Demokritos, Athens, Greece*

*^2^Università Politecnica delle Marche, Dipartimento SIMAU, Via Brecce Bianche 12, Ancona 60131, Italy*

*^3^Faculty of Physics and Center for Nanointegration (CENIDE), University of Duisburg-Essen, 47057 Duisburg, Germany*

*^4^Zavoisky Physical-Technical Institute, FRC Kazan Scientific Center of RAS, 420029 Kazan, Russian Federation*

*^5^ Center for Functionalized Magnetic Materials (FunMagMa), Immanuel Kant Baltic Federal University, Kaliningrad, Russian Federation*

*^6^ SLAC National Accelerator Laboratory- Stanford University,* *Menlo Park, California, USA*

*^7^ IMM-CNR, VII strada 5, 95121 Catania, Italy*

*^8^ Istituto di Struttura della Materia, CNR, Monterotondo Scalo, Roma, Italy*

** Corresponding authors*

*g.giannopoulos@inn.demokritos.gr*

*g.barucca@univpm.it*

**Supplementary Materials section**

**XRF**

XRF allows us to obtain the relative change of stoichiometry across the material library. Measurements were performed at the Si wafer/Cr(10 nm)/ Cu_3_Au(70 nm)/combinatorial-CuAuNi/NiFe(40 nm)/Au(5 nm) sample. The centre position (5,5) is taken as a reference of known stoichiometry, which is obtained by the fact that the deposition rate of each element is known at the centre wafer position. By curve fitting the XRF peaks using a Gaussian-Lorentzian formula, the area of each peak is measured; this area is proportional to the content of each element in the film and relative changes from point to point of the material library may be obtained.

**Figure S1a.** XRF spectrum and the corresponding fitted curves of position (1,5)

**Figure S1b.** XRF spectrum and the corresponding fitted curves of position (5,5). This position is considered of known stoichiometry and is taken as a reference.

**Figure S1c.** XRF spectrum and the corresponding fitted curves of position (9,5).

**Atomic Force Microscopy**

Atomic force microscopy (AFM) has been employed to image the surface morphology of the samples (figure S2). Selected locations across the material library have been imaged and no considerable variation of surface morphology characteristics is observed. The surface roughness and average feature size at each image is shown in table 1.


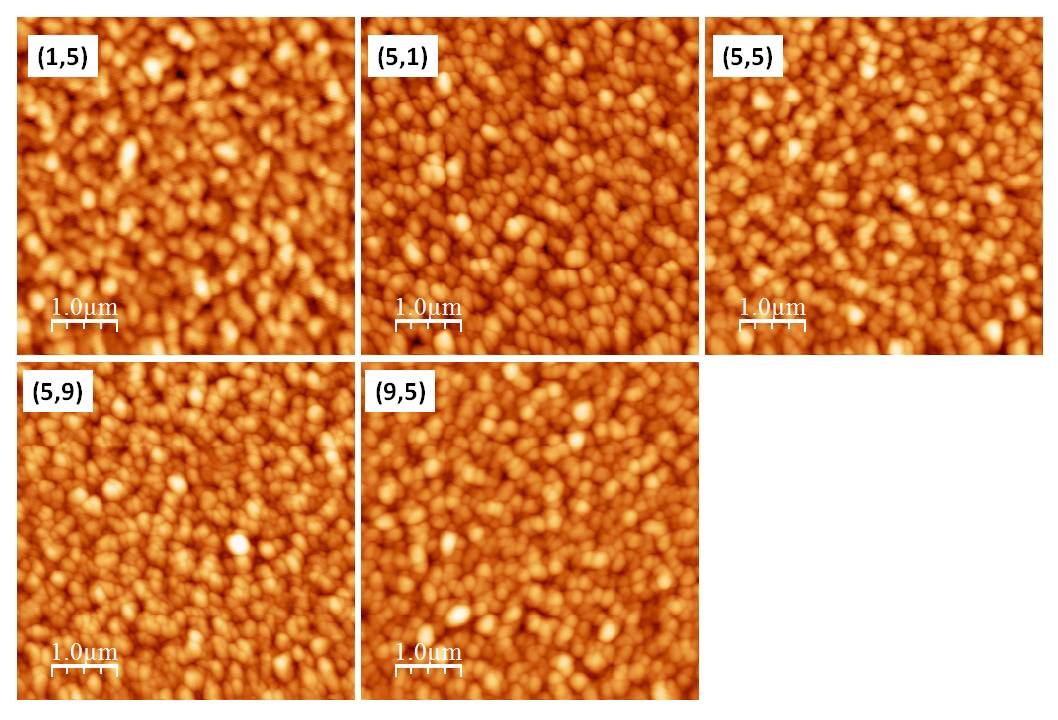


**Figure S2.** AFM images obtained at various positions of the material library. The vertical colour scale is from 0 to 120 nm in all images.

**Table S1.** Surface roughness and average feature size obtained at the images shown in figure3

| Position | (1,5) | (5,1) | (5,5) | (5,9) | (9,5) |
| --- | --- | --- | --- | --- | --- |
| Surface roughness (nm) | 13.8 | 15.3 | 12.6 | 13.6 | 11.5 |
| Average feature size (nm) | 245 | 270 | 278 | 261 | 252 |

**WAXS measurements**

A 2D detector is used to obtain the WAXS spectra. The raw XRD image is mathematically converted to a calibrated XRD image in the diffraction coordinate system, the *Q-γ* plot shown in the Figure S3 a), b) and c). The *x*-axis is the scattering vector *Q* and the *y*-axis measures the azimuthal angle γ between the diffracted beam and the vertical plane containing the incident beam [Fang Ren, Ronald Pandolfi, Douglas Van Campen, Alexander Hexemer, and Apurva Mehta, ACS Comb. Sci. DOI: 10.1021/acscombsci.7b00015]. Diffraction intensity as a function of azimuthal angle probes the grains oriented nearly normal to the surface at the center of the image (see Figure S4 and corresponding discussion) to progressively probing more in-plane grains towards the top and the bottom edges of the image. The noticeable change in the intensity of the L1_0_ (001) diffraction peaks as function of gamma indicates that the L1_0_ films are textured. From these 2D Q−γ images 1D spectra were extracted by averaging a narrow slice (+/- 2 deg) along γ. The Bragg angle (2*θ*) and *Q* are related according to the relationship *Q = 4πsin(θ)/λ*, in which *λ* is the wavelength for the X-ray energy used; thus, a 1D intensity vs. 2*θ* spectrum is generated, Figure S3 d). In the manuscript, the diffraction patterns are converted to 2*θ* values equivalent to Cu k_α_ (1.54 Å) to make our measurements readily accessible to large community of materials scientists accustomed to measuring XRD with Cu k_α_ radiation.


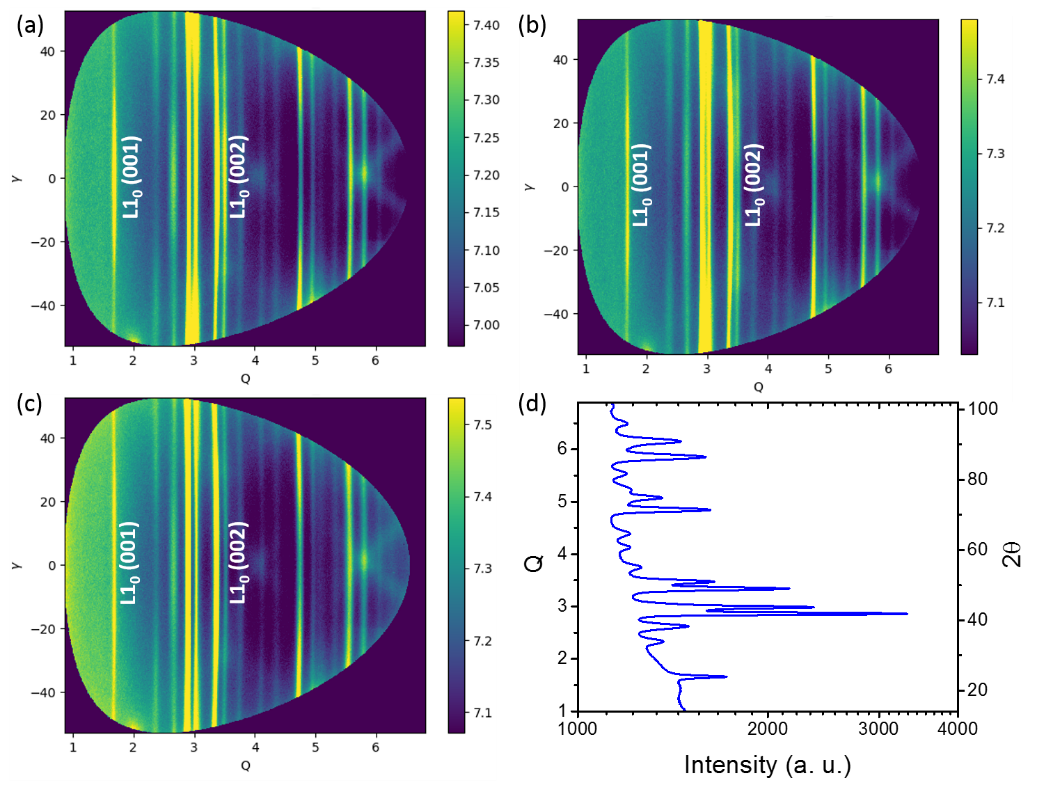


**Figure S3.** *Q-γ* plot of the (a) H, (b) M, and (c) L sample. (d) Representative 1D spectrum of the H sample. Left axis: Q-space. Right axis: Bragg angle space with Cu K_α_ X-ray source.

To identify the WAXS peaks, the ordered Cu_3_Au phase as reported in the ICDD (International Centre for Diffraction Data) n° 351357 was considered, while for the ordered L1_0_-FeNi phase the lattice parameters reported in literature were used [Sho Goto et al.; Synthesis of single-phase L1_0_ – FeNi magnet powder by nitrogen insertion and topotactic extraction; Scientific Reports 7:13216, 2017; International Crystal Structure Database; ICSD#:56386]. Considering that for Cu_3_Au phase the lattice parameter is *a* = 0.37493 nm, while for L1_0_ Fe-Ni phase the lattice parameters are quite different (*a* = 0.3576 - 0.3582 nm; *c* = 0.3589-0.3607 nm), the superstructure reflections of the two phases are easily distinguishable.

The X-ray energy employed during experiments was 12.7 keV and the angle of incidence was α = 4^o^. At this energy, the X-ray wave length is λ = 0.9762 Å and considering the *Q* value for the (001) L1_0_ Fe-Ni peak, a Brag angle θ_B_(001) = 8° is obtained. Easy geometrical considerations allow to affirm that the difference between the Brag and the incident angle corresponds to the angle, β, between the (hkl) planes (producing the diffraction signal at the θ_B_) and the sample surface: θ_B_ – α = β. Therefore, the grains giving rise to the (001) L1_0_-FeNi peak have a β angle equal to 4°. As a consequence, being the *c*-axis perpendicular to the (001) atomic planes, it forms an angle of 4° with respect the perpendicular to the film surface. In a similar way, it is possible to recognize that the (011) and (111) L1_0_-FeNi atomic planes form with the substrate surface an angle of about 7° and 10° respectively and the corresponding grains have the *c*-axis tilted away from the film surface.


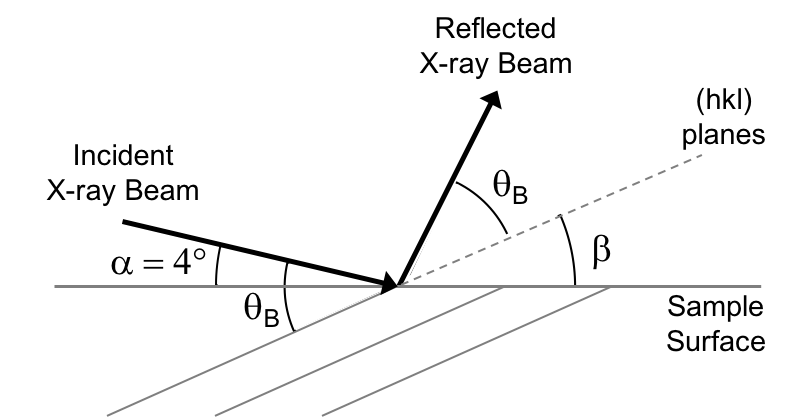


**Figure S4.** Sketch of WAXS measurements geometry

**Transmission Electron Microscopy (TEM)**


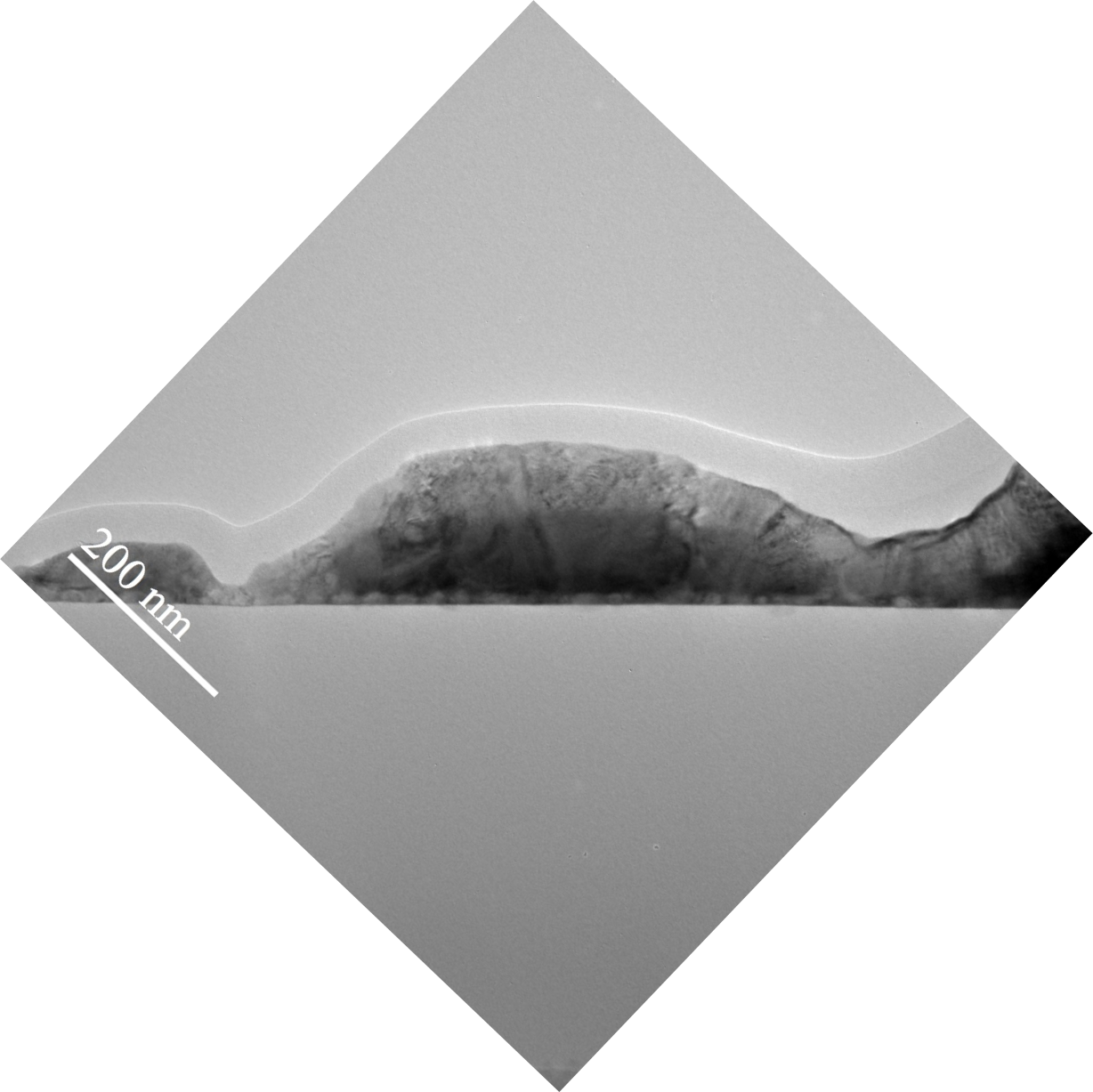


**200 nm**

**Cu_3_Au**

**Figure S5.** Bright field TEM image of the *L*-FeNi sample. The Cu_3_Au layer is clearly visible while the combinatorial layer cannot be distinguished by the FeNi layer suggesting a local epitaxial growth between them.


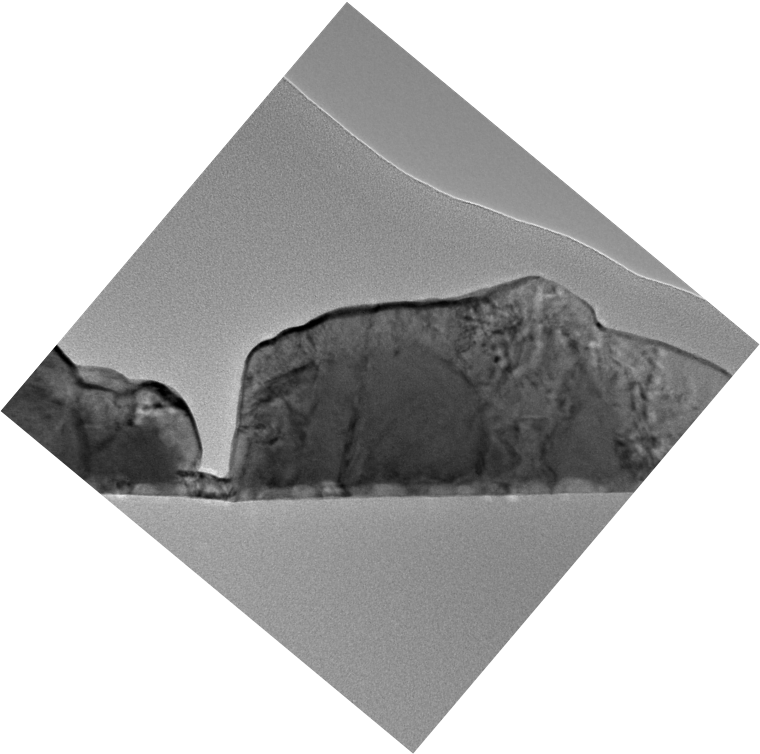


**FeNi**

**100 nm**

**Figure S6.** Bright field TEM image of the *H*-FeNi sample. Different from the previous image, the FeNi layer is clearly visible while the combinatorial layer cannot be distinguished by the Cu_3_Au layer suggesting a local epitaxial growth between them.

**Atomic resolution high-angle annular dark field scanning transmission electron microscopy (HR- HAADF-STEM)**

HR-HAADF STEM images simulation has been performed by using JEMS software (P. A. Stadelmann. JEMS - EMS java version, 2004). The lattice structure of the ordered FeNi phase was obtained as “cif” file in NIMS materials database (http://mits.nims.go.jp).

Figure S6 show two simulated HR-HAADF STEM images of the ordered FeNi phase. The image of Figure S6 a) was obtained considering the electron beam parallel to the [100] direction. The corresponding FeNi cell is shown for explicative reason under the image. In the simulated image the Ni atoms have a larger contrast with respect the Fe atoms, indicating that it is possible to distinguish the two atomic species also if their atomic number is close. The image of Figure S6 b) was obtained considering that the electron beam is tilted of 10° with respect the [100] direction. The rotation was performed around the [001] *c*-axis and under the image the FeNi cell is shown in the corresponding orientation. In this case, it is not possible to resolve atoms in the atomic planes perpendicular to *c*-axis and only alternated strips having different contrast are visible (thin lines between large ones).

**a)**

**b)**


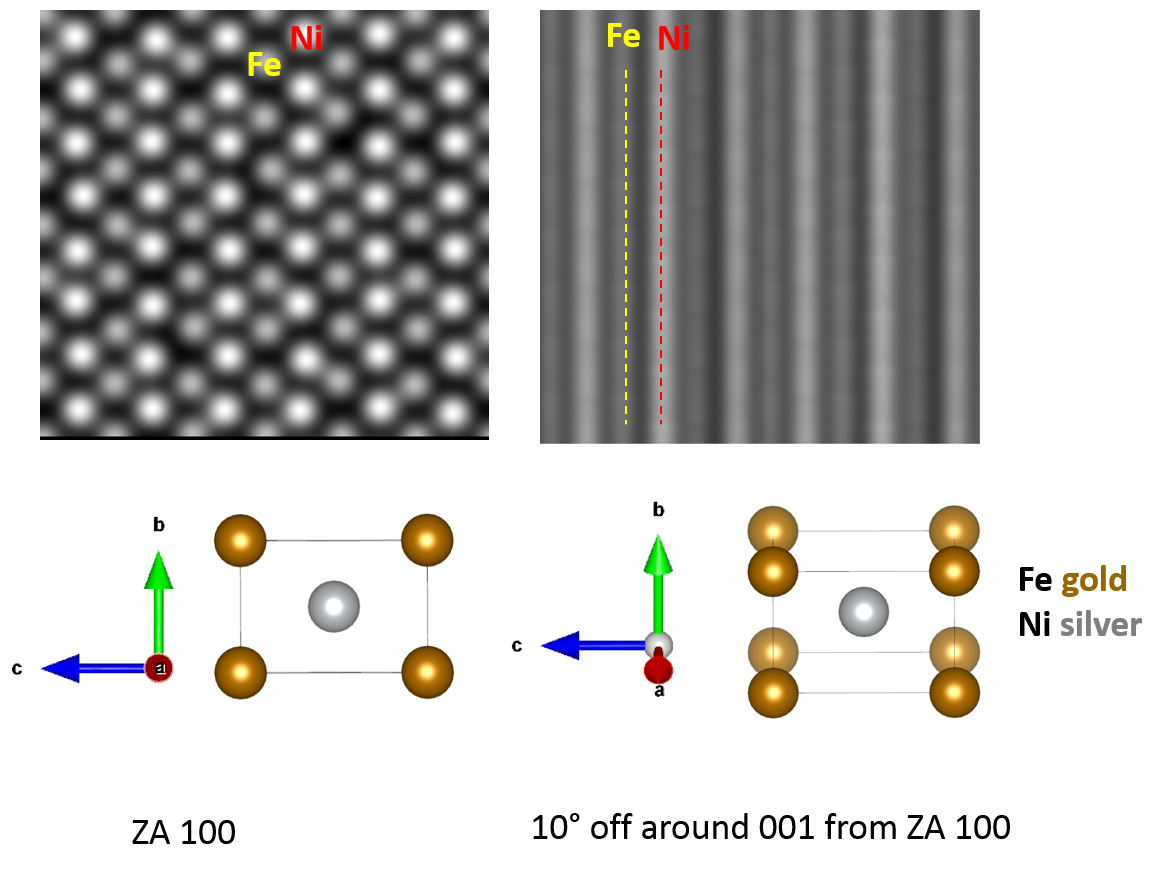


**Figure S7.** Simulated HR-HAADF STEM images of the ordered FeNi phase: a) in [100] zone axis; b) 10° away from [100] zone axis, rotating around the [001] *c*-axis.

This last simulated image shows a contrast very similar to that observed in HR- HAADF STEM images of sample *H*-FeNi. In particular, Figure S7 shows experimental HR- HAADF STEM images of the sample *H*-FeNi. Figure S7 a) is a magnified part of Figure S7 b). The contrast of the light-blue rectangular part of this magnified area was compared with the contrast of the previous simulated image. The graphic reported under the images shows a complete correspondence between the contrast of the experimental and simulated images both for the intensity variation and the distance among the strips. Experimental and simulation results attest the presence of the chemical ordered L1_0_ -FeNi phase in the *H*-FeNi sample.


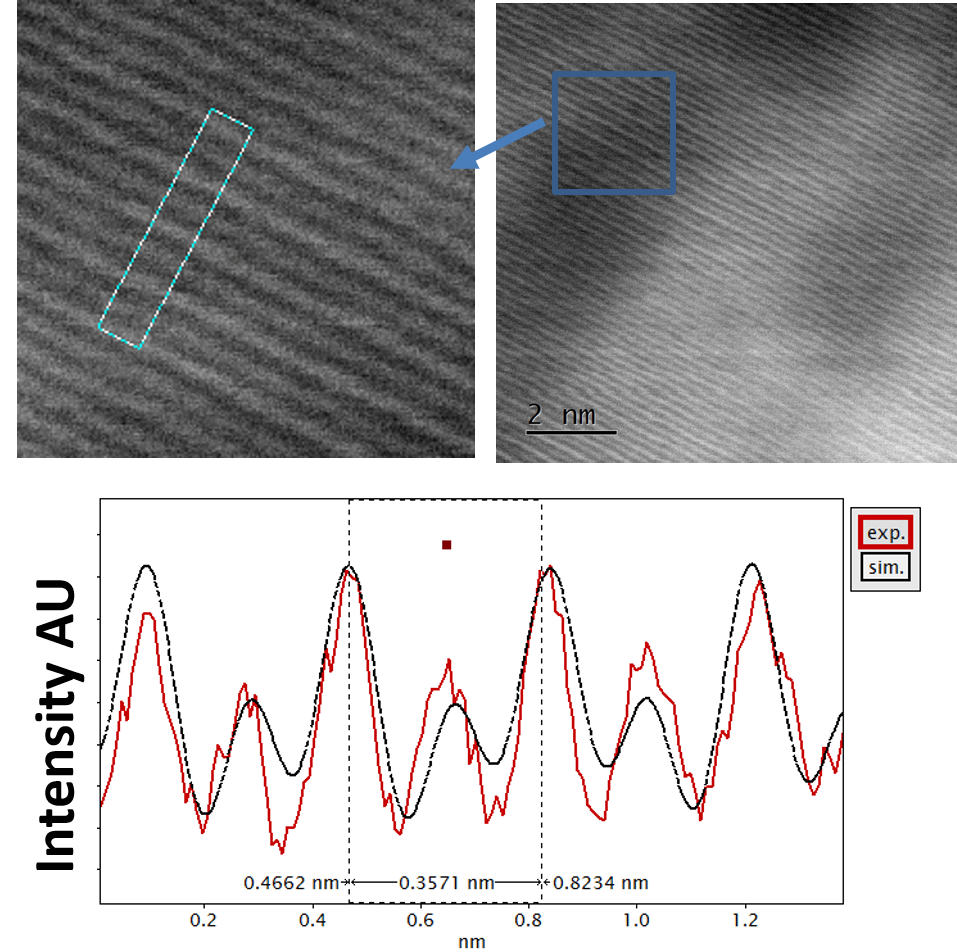


**b)**

**a)**

**Figure S8**: HR-HAADF STEM images of *H*-FeNi sample showing alternated strips with different contrast. Image a) is the magnified area of the sample indicated by the blue square in b). In the graph the contrast of the light-blue rectangular part evidenced in image a) is compared with the contrast of the simulated image of Figure S6 b) revealing a perfect agreement.
